# Confusion with cerebral perfusion pressure in a literature review of current guidelines and survey of clinical practise

**DOI:** 10.1186/1757-7241-21-78

**Published:** 2013-11-21

**Authors:** Vidar Rao, Pål Klepstad, Ole Kristian Losvik, Ole Solheim

**Affiliations:** 1 Department of Neurosurgery, St. Olavs Hospital, Trondheim, Norway; 2 Department of Neuroscience, Faculty of Medicine, Norwegian University of Science and Technology, Trondheim, Norway; 3 Department of Anaesthesiology and Intensive Care Medicine, St. Olavs Hospital, Trondheim, Norway; 4 Department of Circulation and Medical Imaging, Faculty of Medicine, Norwegian University of Science and Technology, Trondheim, Norway; 5 Department of Clinical Medicine, Faculty of Health Science, University of Tromsø, Tromsø, Norway

## Abstract

**Background:**

Cerebral perfusion pressure (CPP) is defined as the difference between the mean arterial pressure (MAP) and the intracranial pressure (ICP). However, since patients with traumatic brain injury (TBI) are usually treated with head elevation, the recorded CPP values depends on the zero level used for calibration of the arterial blood pressure. Although international guidelines suggest that target values of optimal CPP are within the range of 50 – 70 mmHg in patients with TBI, the calibration of blood pressure, which directly influences CPP, is not described in the guidelines.

The aim of this study was to review the literature used to support the CPP recommendations from the Brain Trauma Foundation, and to survey common clinical practice with respect to MAP, CPP targets and head elevation in European centres treating TBI patients.

**Methods:**

A review of the literature behind CPP threshold recommendations was performed. Authors were contacted if the publications did not report how MAP or CPP was measured. A short questionnaire related to measurement and treatment targets of MAP and CPP was sent to European neurosurgical centres treating patients with TBI.

**Results:**

Assessment methods for CPP measurement were only retrieved from 6 of the 11 studies cited in the TBI guidelines. Routines for assessment of CPP varied between these 6 publications. The 58 neurosurgical centres that answered our survey reported diverging routines on how to measure MAP and target CPP values. Higher CPP threshold were not observed if blood pressure was calibrated at the heart level (p = 0.51).

**Conclusions:**

The evidence behind the recommended CPP thresholds shows no consistency on how blood pressure is calibrated and clinical practice for MAP measurements and CPP target values seems to be highly variable. Until a consensus is reached on how to measure CPP, confusion will prevail.

## Introduction

Maintaining an adequate cerebral perfusion pressure (CPP) is crucial in patients with traumatic brain injury (TBI). CPP is defined as the difference between the mean arterial pressure (MAP) and the intracranial pressure (ICP). Aggressive attempts to keep the CPP above 70 mmHg have been reported to be detrimental [1,2], as have CPP levels below 50 mmHg [3]. International guidelines by the Brain Trauma Foundation [4] therefore propose that target CPP should be somewhere between 50 to 70 mmHg, but due to the weaknesses in the existing literature, the optimal CPP after traumatic brain injury is still not settled.

**Figure 1:**
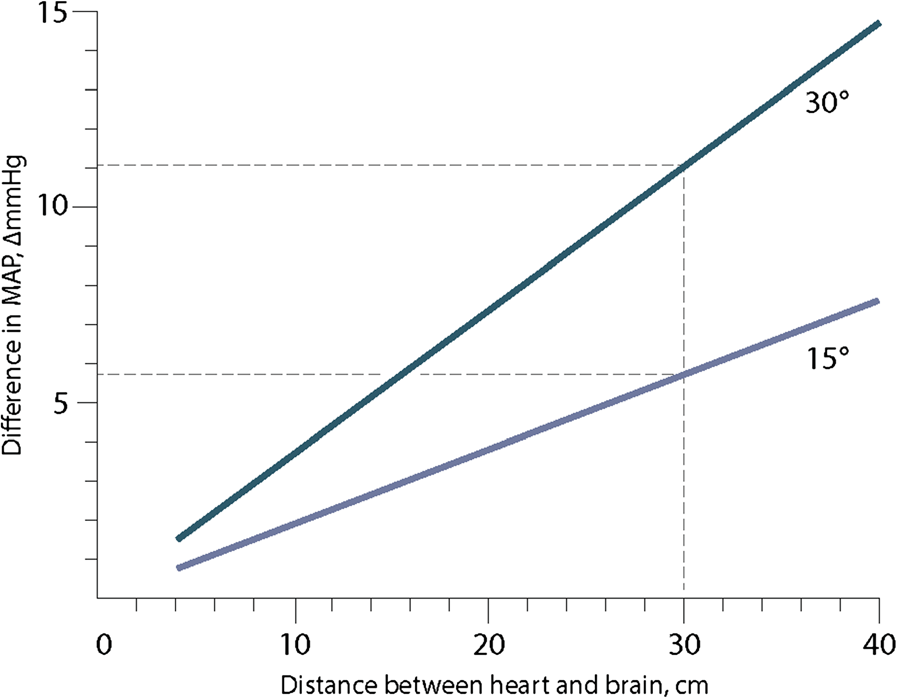
The difference in MAP/CPP (mmHg) depends on the degree of head elevation, as well as the distance between the heart and the head.

Curiously, how to measure MAP and consequently also CPP in the first place, has not been given much attention. Since most patients with TBI are managed with head elevation, the level of zero calibration of the arterial blood pressure will affect the MAP, and hence CPP levels, significantly. Simple trigonometry reveals that in a person with 30 degrees elevation head and 30 cm distance between heart and the head, the difference in measured MAP/CPP levels will be 11 mmHg depending on if the blood pressure transducer is calibrated in the heart or head level (Figure 1).

The aim of this study was to review the literature that the CPP guidelines are based upon, regarding calibration of the blood pressure transducer for measuring MAP and CPP. We also performed a survey of clinical practice with respect to MAP, CPP and head elevation in European centres treating TBI patients.

## Methods

The Brain Trauma Foundation has published recommendations regarding CPP thresholds. As evidence for the present recommendation, a total of 11 studies are listed, of which 6 are classified as new, i.e. published between the years 2000 – 2005. These 11 publications were obtained in full text and reviewed carefully to see if there were any details in the text regarding how the MAP was measured, and even more importantly, at what level the arterial line was calibrated. If this information was not revealed in the publications, we contacted the corresponding author by e-mail and/or mail. Evidence levels and grades of recommendations were assessed according to the classification from Oxford Centre for Evidence-Based Medicine. We have not attempted to do a systematic literature search and review of the available literature not included in the Brain Trauma Foundations guidelines.

A short questionnaire was sent to European centres treating patients with TBI. The survey was conducted by using an Internet-based survey tool (SurveyMonkey Inc., Palo Alto, CA, USA, ( http://www.surveymonkey.com)). In February 2013 a short questionnaire (Table 1) was sent by e-mail to the head of 309 European neurosurgical centres treating patients with brain injuries. The e-mail addresses were obtained by contacting the national neurosurgical societies in each respective country. A reminder e-mail was sent to all non-responders after 1 week and 4 weeks, before the survey was closed after 8 weeks. All answers were managed anonymously.

**Table 1:** The questionnaire and answers

**Questions**	**Response alternatives**	**Answers (%)**
**1. In patients with severe traumatic head injuries treated with intensive care, what are the cerebral perfusion thresholds at your hospital (i.e. what CPP levels are you aiming at)?**	**A: 50 – 70 mmHg**	**A: 40,4%**
	**B: >60 mmHg**	**B: 56,1%**
	**C: Other**	**C: 3,5%**
**2. What is the level of head elevation used in patients with severe head injuries treated with intensive care at your hospital?**	**A: 0 degrees**	**A: 1,7%**
	**B: 15 degrees**	**B: 25,9%**
	**C: 30 degrees**	**C: 67,2%**
	**D: Other**	**D: 5,2%**
**3. Cerebral Perfusion Pressure = Mean Arterial Pressure – Intracranial Pressure (CPP = MAP – ICP). However, at what level is the MAP calibrated to zero for continuous CPP monitoring?**	**A: Head level**	**A: 36,2%**
	**B: Heart level**	**B: 62,1%**
	**C: Other**	**C: 1,7%**

The statistical analysis and graphs were performed using IBM Statistical Package for the Social Sciences (SPSS) V.21. A Pearson Chi-square test was used to investigate the relationship between desired CPP-thresholds and level of arterial line calibration.

## Results

### Review of the literature

The Brain Trauma Foundation has published recommendations regarding CPP thresholds. The list of evidence supporting this recommendation is presented in Table 2, and consists of 11 studies published between 1987 – 2005. In only three of the listed publications, information about MAP calibration was provided. By contacting the corresponding authors, we were able to get this information in an additional three publications. These six publications had quite different approaches to calibrate the MAP: Two of the six authors reported that they referenced the MAP (and ICP) at head level. One author referenced the MAP transducer at heart level. One levelled "…ICP and MAP in relation to the head tilt". One measured MAP in the mid-axillary line, whereas one had the patients in a supine position, and hence the MAP and ICP were measured at the same level. Despite persistent attempts to contact the authors we were not able to obtain information regarding MAP assessments from the remaining five publications.

**Table 2:** Evidence table for cerebral perfusion pressure recommendations

**Reference**	**MAP calibration level**^**1**^	**Main findings**	**Level of evidence**^**2**^	**Grade of recommendation**^**2**^
Changaris et al., 1987 [5]	*MAP measured at heart level	All patients with CPP < 60 mmHg on the second post-injury day died. More patients had a good outcome when CPP > 80 mmHg.	III	C
McGraw, 1989 [6]	NA	The likelihood of good outcome was higher, and mortality lower when CPP > 80 mmHg.	III	C
Rosner and Daughton, 1990 [7]	Supine position. Systemic ABP, transducer at same level as ICP	CPP actively kept >70 mmHg gave the same morbidity rates as previous methods.	III	C
Cruz, 1998 [8]	"ICP and MAP levelled in relation to the head tilt"	Monitoring cerebral extraction of oxygen in conjunction with CPP gave better outcome than when CPP is managed alone.	III	C
Robertson et al., 1999 [2]	MAP measures at the same level as ICP	CPP > 70 mmHg increased the risk of ARDS.	II	B
Juul et al., 2000 [9]	*Arterial line, head level	CPP > 60 mmHg had no influence on outcome.	III	C
Contant et al., 2001 [1]	NA	Increased risk of ARDS when CPP > 70 mmHg.	III	C
Andrews et al., 2002 [3]	NA	Low CPP and hypotension were predictors of death and poor outcome.	III	C
Clifton et al., 2002 [10]	NA	Poor outcome was associated with a CPP < 60 mmHg. No benefit by maintaining CPP > 70 mmHg.	III	C
Steiner et al., 2002 [11]	NA	Optimal CPP for each patient was calculated. Patients whose CPP varied above or below had a worse outcome.	III	C
Howells et al., 2005 [12]	*MAP measured in mid-axillary line	Patients with intact auto-regulation had better outcomes with CPP > 70 mmHg. Patients with defect auto-regulation had better outcome with ICP targeted care.	III	C

^1^Information about how the arterial line was calibrated to measure the mean arterial blood pressure (MAP) was either found in the publication, or obtained from the corresponding author (*). NA: Not available. ^2^Evidence levels and grades of recommendation, adapted from the Oxford Centre for Evidence-Based Medicine for the UK National Health Service.

The main conclusions regarding CPP-levels from the available evidence is presented in Table 2, together with evidence levels and grades of recommendation. All publications except one are labelled as level III evidence and result in grade C recommendations, while one paper provides level II evidence and constitutes a grade B recommendation.

### What is clinical practice?

The questionnaire was sent to the head of the neurosurgical department in 309 European hospitals. If the respondent was unable to answer the questions, they were instructed to forward the e-mail to the person in charge of neuro-intensive care at their centre (neurosurgeon or anaesthesiologist). After two reminder e-mails to the non-respondents, a total of 58(19%) centres responded to the survey.

Clinical practice for target CPP values, degree of head elevation and MAP calibration levels varied between European neurosurgical centres (Table 1). 56% reported that they aimed for a CPP above 60 mmHg, whereas 40% sought to keep CPPs between 50 and 70 mmHg. All respondents except one routinely use some degree of head elevation. The majority (67%) use 30 degrees of head elevation. There were also different routines regarding what level the MAP transducer was calibrated to zero: 62% calibrate at heart level, while 36% calibrate at head level. One respondent initially calibrates at heart level, but recalibrates at head level when ICP rise above 20 mmHg.

There was no significant correlation between the desired CPP targets and routines for MAP calibration levels among the respondents, p = 0,51 (Table 3).

**Table 3:** The relationship between response in the survey when it comes to desired CPP thresholds and MAP calibration level

		**MAP calibration (n)**		**Total (n)**
		**Head level**	**Heart level**	
**CPP thresholds (n)**	50-70 mmHg	8	16	24
	>60 mmHg	13	18	31
Total (n)		21	34	55

Pearson Chi-square p = 0.51.

## Discussion

Measurements of and interventions to obtain optimal CPP is a cornerstone in neuro-intensive care. However, routines on how to measure MAP will affect observed CPP levels profoundly. Unfortunately, we find that studies behind the recommended CPP thresholds often do not elaborate on how MAP and CPP are measured. Additionally, methods of MAP and CPP measurements vary considerably in the studies where this information was available. The only level II evidence study cited in respect to CPP in the TBI guidelines measured MAP and ICP at the same level [2]. This randomised study from 1999 compared an ICP-targeted protocol and a CPP-targeted protocol and reports that the risk of cerebral ischemia was 2.4-fold greater with the ICP-targeted protocol, but that the risk of ARDS was a 5-fold greater with a CPP-targeted protocol. Median CPP levels were >70 mmHg in both protocols, but somewhat lower in the ICP-targeted group. This single study is the basis for the level II recommendation against CPP > 70 mmHg in the Brain Trauma foundation guidelines. In addition to resting on limited evidence from few studies, the recommended CPP target values can be questioned since there is no consensus on how to measure CPP in the first place. Indeed, our small survey among neurosurgical departments further demonstrates that clinical practice is highly variable, both in terms on how to measure CPP and when to intervene.

A limitation of the study was the low response rate in the survey, but it was somewhat as expected when comparing to other internet based surveys [13]. This raises some caution against the interpretation of the distribution of responses within each category. Nevertheless, the 58 respondents give a clear answer of that practices vary between neurosurgical centres related to the measurement of CPP and treatment of TBI patients. As it is very unlikely that only centres with a varied practice answered the survey, a higher response rate would not change the fact that there are different routines. The principles of validity and reliability are fundamental cornerstones of all scientific and clinical measures. While the low number of studies and the low evidence level published limit the validity of CPP when it comes to the clinical implication (e.g. target values), the diversity in how CPP is measured both in the literature and in clinical practice greatly limits the reliability. A shortness in both reliability and validity is clearly problematic for any measure.

In conclusion, the methods for CPP measurements in studies used for development of TBI guidelines are often not reported. Studies that report how CPP is obtained use various methods. Clinical practice related to the measurement and treatment of CPP varies between neurosurgical centres. Until a consensus is reached on how to measure CPP, confusion will prevail.

## Abbreviations

TBI: Traumatic brain injury; MAP: Mean arterieal pressure; ICP: Intracranial pressure; CPP: Cerebral perfusion pressure.

## Competing interests

None of the authors have any competing interest or financial disclosures to declare.

## Author’s contributions

VR participated in the design of the study, the review of literature, data collection/processing/interpretation and drafting the manuscript. PK contributed in the design of the study, interpretation of data and drafting the manuscript. OKL participated in the design of the study, creation of figures, interpretation of data and drafting the manuscript. OS participated in the conception and design of the study, the review of literature, data processing/interpretation and drafting the manuscript. All authors read and approved the final manuscript.
